# Application of a Rapid and Efficient Quantitative Analysis Method for Traditional Chinese Medicines: The Case Study of Quality Assessment of *Salvia miltiorrhiza Bunge*

**DOI:** 10.3390/molecules18066919

**Published:** 2013-06-13

**Authors:** Wen-Guang Jing, Jun Zhang, Li-Yan Zhang, Dong-Zhe Wang, Yue-Sheng Wang, An Liu

**Affiliations:** 1 Institute of Chinese Materia Medica, China Academy of Chinese Medical Sciences, Beijing 100700, China; 2 Medicine College of Guiyang College of Traditional Chinese Medicine, Guiyang College of Traditional Chinese Medicine, Guiyang 550002, China

**Keywords:** *Salvia miltiorrhiza Bunge*, reference extractive, quantitative analysis, high-performance liquid chromatography, quality assessment

## Abstract

A reference extractive, containing multiple active known compounds, has been considered to be an alternative to individual reference standards. However, in the Chinese Pharmacopoeia (ChP) the great majority of reference extractives have been primarily used for qualitative identification by thin-layer chromatography (TLC) and few studies on the applicability of reference extractives for quantitative analysis have been presented. Using *Salvia miltiorrhiza Bunge* as an example in this paper, we first present a preliminary discussion on the feasibility and applicability of reference extractives for the quantitative analysis of TCMs. The reference extractive of *S. miltiorrhiza Bunge*, comprised of three pharmacological marker compounds, namely cryptotanshinone, tanshinone I and tanshinone IIA, was prepared from purchased *Salvia miltiorrhiza Bunge* by extraction with acetone under reflux, followed by silica gel column chromatography with stepwise elution with petroleum ether-ethyl acetate (25:1, v/v, 4.5 BV) to remove the non-target components and chloroform-methanol (10:1, v/v; 3 BV) to yield a crude reference extractive solution. After concentration, the solution was further purified by preparative reversed-phase HPLC on a C_18_ column with isocratic elution with 77% methanol aqueous solution to yield the total reference extractive of *S. miltiorrhiza Bunge.* Thereafter, the reference extractive was applied to the quality assessment of *S. miltiorrhiza Bunge* using high-performance liquid chromatography (HPLC) coupled with diode array detection (DAD). The validation of the method, including linearity, sensitivity, repeatability, stability and recovery testing, indicated that this method was valid, reliable and sensitive, with good reproducibility. The developed method was successfully applied to quantify seven batches of samples collected from different regions in China and the results were also similar to those obtained using reference standards, with relative standard deviation (RSD) <3%. Preparation of a reference extractive of *S. miltiorrhiza Bunge* was significantly less expensive and time consuming than preparation of a corresponding reference standard. Quantitative analysis using a reference extractive was shown to be simple, low-cost, time-saving and practical, with high sensitivity and good stability; and is, therefore, a strong alternative to the use of reference standards.

## 1. Introduction

Due to the increasing global interest in Traditional Chinese Medicines (TCMs), a legal foundation for establishing the scientific and appropriate quality standards for TCMs is both essential for the modernization and internationalization of TCMs and necessary to satisfy the urgent need for long-term studies to ensure their clinical safety and efficacy [1].

In the past several decades, many changes have taken place in the development of TCMs quality standards, especially due to the wide application of modern analytical techniques. Chromatography coupled with spectrometric detectors such as the diode array detector (DAD), have provided previously inaccessible information that has been helpful in distinguishing false herbs and evaluating the quality of TCMs [2,3]. Because the quantitative determination of a single marker compound was increasingly important in the quality control of TCMs, HPLC had been adopted for the quantitative analysis of 1,206 Chinese herbs in the 2010 edition of the *Chinese Pharmacopoeia*. However, problems may arise due to a lack of adequate reference standards, high cost of detection techniques and instability of some reference standards. Moreover, the preparation and calibration of the reference standards is time-consuming, labor intensive and expensive because of the complexity of the chemical composition of TCMs. Additionally, the determination of a single marker compound was far from sufficient for comprehensively evaluating the quality of TCMs. Consequently, the simultaneous determination of multi-active components in TCMs had become the primary aim of research in the field of quality evaluation of TCMs.

A reference extractive is a substance containing known contents of multiple active compounds. To date, 16 reference extractives had been recorded in the *Chinese Pharmacopoeia* (2010 edition), such as the total extractive of *Ginkgo biloba*, the total saponin extractive of *Dioscorea pathaica* and the volatile oil of *Illicium verum Hook f*. A great majority of the reference extractives were primarily used for thin-layer chromatography (TLC). TLC identification using reference extractives has a much higher specificity than the use of single reference standards and is particularly useful as a replacement for reference standards that are unstable or difficult to obtain. To a certain extent, it is equivalent to a qualitative fingerprint. Based on our literature survey, few reports focusing on the applicability and feasibility of reference extractives used for quantitative analysis of TCMs have been presented and published [4]. If the reference extractive could be used for both qualitative identification and quantitative analysis, then this measure would be equivalent to multi-component quality evaluation, which would certainly be beneficial for the quality control of TCMs. The most fundamental and essential requirement for a reference extractive suitable for use in quantitative analysis was that it had to have a stable and reproducible chromatographic behavior, which also requires that the composition of the compounds in the reference extractive be stable as well. Accordingly, the reference extractives should contain known contents of each compound in proportions that are fairly fixed and close to those found in the crude herbs. In this way, the reference extractive could be used for both the identification of chromatography peaks and quantitative analysis.

*S. miltiorrhiza Bunge* is an important and representative TCM known as “Danshen”. Danshen root and its preparations, such as compound Danshen tablets, compound Danshen dripping pills and Danshen injections, are used in the prevention and treatment of coronary heart disease, cerebrovascular disease, hyperlipidemia, hepatitis, chronic renal failure, dysmenorrhoea and neurasthenic insomnia [5,6,7]. Pharmacological studies of *S. miltiorrhiza Bunge* have revealed a variety of biological activities, including anti-tumor, anti-inflammatory, antimicrobial, anti-viral and antioxidant effects [8,9,10,11]. Thus, the worldwide consumption of *S. miltiorrhiza Bunge* and its extractives has been increasing due to its comprehensive pharmacological activities. As a result, quality control of *S. miltiorrhiza Bunge* is very important, especially for its clinical efficacy and safety. According to the current literature reports, several methods including HSCCC [12], HPTCL [13], NAPE [14] HPLC [15,16,17], and HPLC-MS [18,19,20,21] have been established for the quality control of *S. miltiorrhiza Bunge*, and substantial bioactive components such as danshensu, protocatechuic aldehyde, salvianolic acid B, cryptotanshinone, tanshinone IIA and tanshinone I were separated, identified and used for quantitative analysis. In China, quality control of *S. miltiorrhiza Bunge* was performed by the determination of tanshinone IIA and salvianolic acid B as the marker compounds using HPLC [22]. However, studies show that nonpolar compounds, such as tanshinone I and cryptotanshinone, were also beneficial active compounds responsible for good biological activities, with anti-tumor properties [23,24]. Since many of the existing quality control methods require a large amount of reference standard, and these standards are difficult to separate and expensive to generate, this places a significant economic burden on the quality control processes associated with production of marker compounds. Accordingly, it was particularly important and imperative to develop a reference substance which could be substituted single reference for quantitative analysis and could be easily and rapidly prepared as well.

Therefore, taking *S. miltiorrhiza Bunge* as an example, the preparation of the reference extractive using tanshinone IIA, tanshinone I and cryptotanshinone as the marker compounds is described in detail. Moreover, the quantitative analysis method using the prepared reference extractive has been tentatively applied to the quality assessment on the crude herbs of *S. miltiorrhiza Bunge* by comparison with the single reference standard. The final goal of this work was to develop a rapid and efficient quantitative analysis method by using reference extractive, which could be used for quality control of more traditional Chinese herbs, especially for the lack or high price of reference standards, ultimately contributing to the development of TCMs.

## 2. Results and Discussion

### 2.1. Selection of Chromatographic Conditions

To obtain the optimal elution conditions for the separation and determination of the marker compounds, various isocratic gradients of aqueous solutions and acetonitrile at a flow rate of 1.0 mL/min were investigated. In the optimal isocratic elution, the peaks of the three compounds could be well separated with aqueous phosphoric acid (0.02%, v/v) and acetonitrile (58%, v/v) as the mobile phase. The UV absorption maxima for cryptotanshinone, tanshinone I and tanshinone IIA was located at 263, 245 and 270 nm, respectively. Hence, the characteristic chromatographic patterns were obtained by setting the detection wavelength to 270 nm.

### 2.2. Selection of Extracting Condition of *S. miltiorrhiza* Bunge

Extraction solvent and method were two of the most important aspects in the extraction process studied in our work. With the content of tanshinone IIA, tanshinone I, and cryptotanshinone as the index, some extracting methods (M1~M5) reported in the literature [25,26] were preliminarily investigated. In addition, a comparison of the total contents of the extractives was also discussed. Table 1 shows that the peak areas of these three compounds obtained by reflux extraction with 95% ethanol (M4) and acetone (M5) were very similar and significantly higher than those seen with the other three methods M1~M3. Although the extraction content of M5 was apparently less than that of the other methods, the low contents of the non-target substance could be highly beneficial to the purification of the reference extractive. As a result, based on the peak area of these three compounds and their fat-soluble properties, M5 was preferable and acetone was selected as the optimal extraction solvent. 

**Table 1:** Comparison of extraction methods (n = 3).

Extraction methods ^a^		Mean sample weight (g)		Peak area			Total Contents (%)
				Cryptotanshinone	Tanshinone I	Tanshinone IIA	
M1	2.0001		181353		655246	1888524	4.06
M2	2.0002		1213992		855590	2806532	5.99
M3	2.0002		ND ^b^		333004	1426994	3.60
M4	2.0001		1769824		933538	2958279	6.12
M5	2.0000		1654504		841142	3494657	1.06

^a^ **M1:** reflux extraction with 95% ethanol for 1 h, followed by reflux extraction with water for 1 h and elution with 3% Na_2_CO_3_ aqueous solution; **M2:** reflux extraction with 95% ethanol for 1 h followed by reflux extraction with water for 1h; **M3:** reflux extraction with 95% ethanol for 1 h followed by reflux extraction with 3% Na_2_CO_3_ aqueous solution for 1 h; **M4:** reflux extraction with 95% ethanol for 2 h. **M5:** reflux extraction with acetone for 2 h; ^b^ ND: not detected.

To conserve the expensive solvent, the same method using eight times the volume of the menstruum was compared with M5. The results (Table 2) indicated that the content of the maker compounds using both volumes showed little differences, with RSD of 1.09, 1.42, and 1.49, respectively. Consequently, the extracting solvent volume was set at eight times, not only because of economy but also because complete extraction was possible. 

**Table 2:** A comparison of the use of different volumes of the menstruum (n = 3).

Different volumes of the menstruum	Cryptotanshinone (%)	Tanshinone I (%)	Tanshinone IIA (%)
M5	0.1506	0.1412	0.1871
M6	0.1483	0.1384	0.1832
RSD (%)	1.09	1.42	1.49

**M5**: 2 g of the *S. miltiorrhiza Bunge* powder was extracted with 50 mL of refluxing acetone for 2 h, equal to 25 times the acetone. **M6**: 2 g of the powder was extracted with 8 times the amount of refluxing acetone (16 mL) for 2 h.

### 2.3. Removing Non-Target Components with Silica Gel Column Chromatography

The mixed powder which contained silica and crude extract was evaporated to dryness, pulverized and chromatographed on a silica gel column (column volume: 50 mL) with a petroleum ether (PE)- ethyl acetate (EtOAC) system as the eluting solvent. 

**Figure 1:**
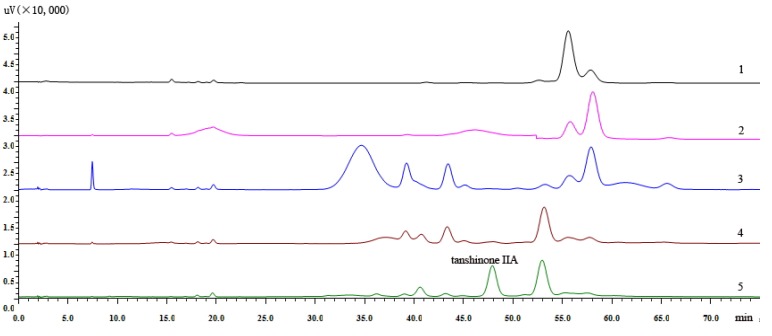
(1) Liquid chromatogram of elution to the 2.5 BV with PE-EtOAC (25:1, v/v). (2) Liquid chromatogram of elution to the 3.0 BV. (3) Liquid chromatogram of elution to the 3.5 BV. (4) Liquid chromatogram of elution to the 4.0 BV. (5) Liquid chromatogram of elution to the 4.5 BV.

Comparison of the 95:5 (v/v), 90:10 (v/v), 25:1 (v/v) ratios of the petroleum ether-ethyl acetate system in combination with TLC and HPLC analysis, showed that the 25:1 (v/v) ratio of the PE-EtOAC was the optimal eluting solvent. The mixed powder was subjected to silica gel column and eluted successively with PE-EtOAC (25:1, v/v) for 4.5 BV while collecting 0.5 BV each time to remove non-target components. It can be seen from Figure 1 that tanshinone IIA, which had smaller polarity was eluted from the silica gel column when eluting at 4.5 BV.

In order to rapidly elute the maker compounds from the silica gel and ensure less impurities after eluting with 4.5 BV of PE- EtOAC (25:1, v/v), elution using PE-EtOAC (3:1, v/v) and chloroform (CHCl_3_)-methanol (MeOH) (10:1, v/v) were compared. Meanwhile, the elution effect and expenditure of the eluents were also taken into account for consideration. Complete elution of three compounds from the silica gel column when using chloroform-methanol required 4 BV (10:1, v/v), while elution of these compounds required 14 BV when using PE-EtOAC (3:1, v/v). The use of CHCl_3_-MeOH (10:1, v/v) for elution with 3 BV was preferable to be applied to the further elution of the target components from the silica gel column.

### 2.4. Purification Process of the Reference Extract Using Prep-HPLC

**Figure 2:**
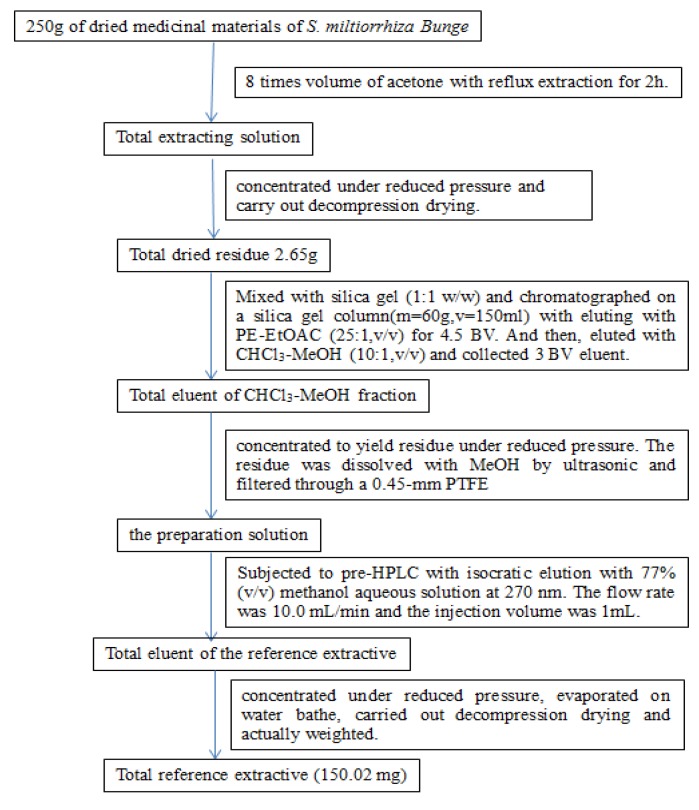
Total technological process of the reference extractive of *S. miltiorrhiza Bunge.*

The combined eluent of the CHCl_3_-MeOH (10:1, v/v) fraction was concentrated under reduced pressure to give a residue which was subjected to MeOH with ultrasonication and filtered through a 0.45 mm PTFE filter to obtain the prepared solution. The solution was concentrated and loaded into a prep-HPLC system coupled with a UV detector. It was noteworthy that the separation between tanshinone I and cryptotanshinone was difficult due to their similar retention time, which directly leads to correspondingly higher prices of both of these two reference standards. However, during the preparation of the reference extractive, baseline separation was not required, and the mixture of both compounds could be collected without additional monomer separation. The total technological process according to the procedures mentioned above was repeatedly validated and is summarized in Figure 2.

### 2.5. Identification of the 3 Marker Compounds in the Reference Extractive Using HPLC-ESI-IT-MS^n^

**Figure 3:**
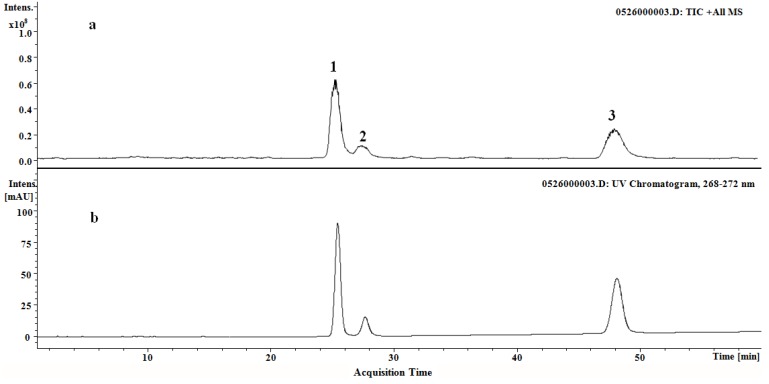
(**a**) TIC chromatogram in positive ion mode. (**b**) HPLC-DAD chromatogram at 270 nm (**1**) Compound **1** at 24.7 min. (**2**) Compound **2** at 27.1 min. (**3**) Compound **3** at 47.1 min.

To identify the main corresponding compounds in the reference extractive, subsequent HPLC-ESI-IT-MS^n^ analysis was performed in positive mode. As illustrated in the total ion chromatogram (Figure 3), the main compounds were all detected in the reference extractive. 

**Figure 4:**
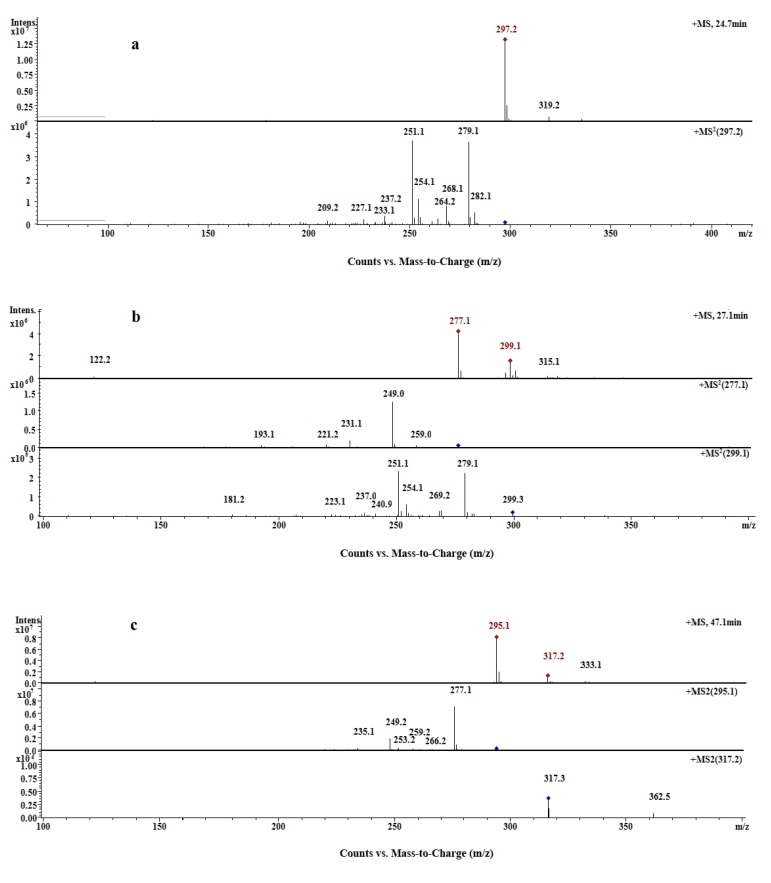
(**a**) MS/MS spectra of compound **1** at 24.7 min. (**b**) MS/MS spectra of compound **2** at 27.1 min. (**c**) MS/MS spectra of compound **3** at 47.1 min.

Compounds **1**-**3** were characterized based on their elution times, UV absorption and MS fragmentation behavior as cryptotanshinone, tanshinone I and tanshinone IIA, respectively. The compounds produced [M+H]^+^ ions at *m/z* 297, 277, 295, respectively, suggesting that the molecular weights of these three compounds were 296, 276 and 294, respectively. Adduct ions [M+Na]^+^ could be observed occasionally in their MS spectra (Figure 4). Compound **1** was identified as cryptotanshinone. Fragmentation in the positive mode MS/MS spectrum focused on the precursor ion [M+H]^+^ (*m/z* 297) which showed peaks corresponding to the successive loss of two molecules of H_2_O and give the product ions at *m/z* 279 [M+H−H_2_O], *m/z* 251 [M+H−2H_2_O]. Some other typical fragment ions at *m/z* 282 [M+H−CH_3_]^+^ and *m/z* 268 [M+H−C_2_H_4_]^+^ can also be observed in MS^2^ spectrum. These findings are consistent with the literature data [27].

Compound **2** produced the [M+H]^+^ ion at *m/z* 277, [M+Na]^+^ at *m/z* 299 and further yielded a series of fragment ions including [M+H−CO]^+^ at m/z 249, [M+H−H_2_O]^+^ at *m/z* 259, [M+H−H_2_O−CO]^+^ at *m/z* 231 and [M+H−CO−2CO]^+^ at *m/z* 193. By comparison with an authentic standard and literature reports [28], compound **2** was characterized as tanshinone I.

The precursor ion [M+H]^+^ at *m/z* 295 gave prominent product ions at *m/z* 277 and 249, generated from the successive loss of H_2_O and CO. Other characteristic ions including [M+H−CHO]^+^ at *m/z* 266, [M+H−H_2_O−CO]^+^ at *m/z* 235 could also be detected in positive mode. By referring to literature data [28], compound **3** was identified as tanshinone IIA, which gave a maximum absorption at about 270 nm in the DAD spectrum. 

### 2.6. Quantitative Calibration Analysis of the Three Compounds in the Reference Extractive Using HPLC with the Corresponding Reference Standards

**Figure 5:**
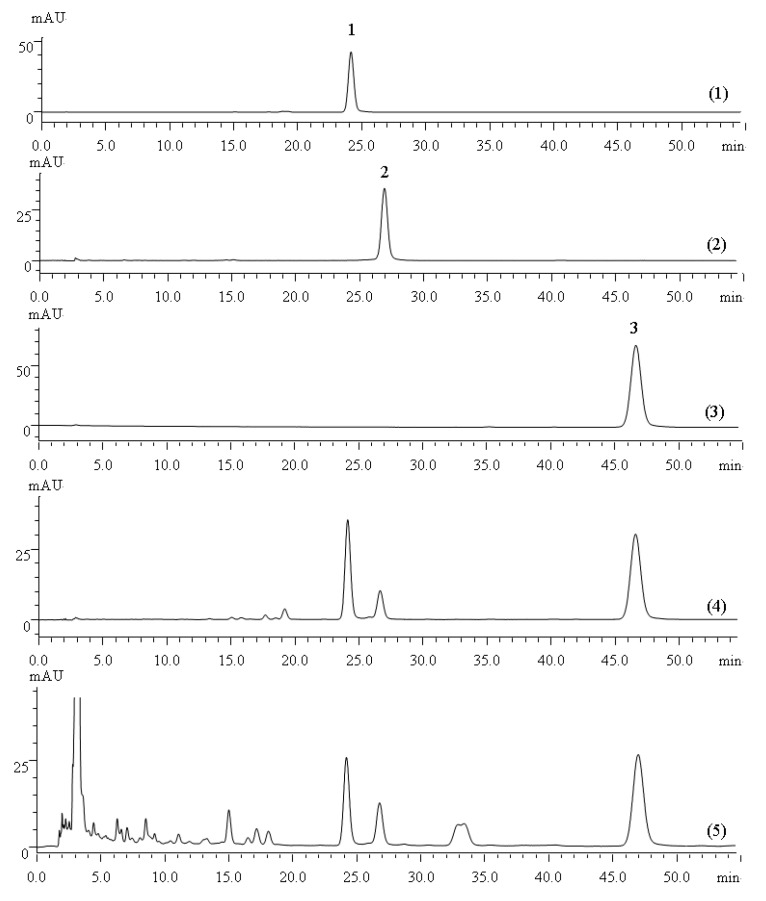
(**1**) HPLC chromatogram for the cryptotanshinone reference standard; (**2**) HPLC chromatogram for the tanshinone I reference standard; (**3**) HPLC chromatogram for the tanshinone IIA. reference standard; (**4**) HPLC chromatogram for the *S. miltiorrhiza Bunge* reference extractive; (**5**) HPLC chromatogram for the *S. miltiorrhiza Bunge* crude sample. Peak **1**: cryptotanshinone; Peak **2**: tanshinone I; Peak **3**: tanshinone IIA.

The HPLC chromatograms of the quantification of tanshinone IIA, tanshinone I and cryptotanshinone in the reference extractive of *S. miltiorrhiza Bunge* and crude samples are shown in Figure 5. Method validation for the assay of the three marker compounds was performed, after which the HPLC conditions were carefully optimized, including the mobile phase and detection wavelength. The validation results (calibration curve, precision and repeatability) are listed in Table 3. Good linearity, injection precision, stability and repeatability (RSD < 2%) were demonstrated for these three compounds. 

**Table 3:** Regression, precision, repeatability and stability of the three compounds in the *S. miltiorrhiza Bunge* reference extractive.

Analyte	Regression equation	*r*^2^	Linear range (*μ*g/mL)	Precision (RSD, %, *n* = 6)	Repeatability (RSD, %, *n* = 5)	Stability (RSD,%, *n* = 8)	Total amount (RSD, %, *n* = 3)
Cryptotanshinone	Y = 34464.36X + 2.03	0.9999	3.88~38.80	0.02	0.26	0.08	0.40
Tanshinone I	Y = 17574.55X + 1.31	0.9999	4.41~44.08	0.02	0.87	0.84	1.38
Tanshinone IIA	Y = 48224.6X + 0.87	0.9999	3.99~39.96	0.02	0.48	0.15	0.73

A methanol stock solution containing the standard samples was prepared and diluted to a series of appropriate concentrations for the construction of the calibration curves. Six concentrations of the mixed standard solution were injected in triplicate, and their regression equations were calculated in the form of Y = A × X + B. The intra-day precision was determined by analyzing the six replicated calibration samples on the same day. The RSD was taken as a measure of precision. Stability was tested with an extractive solution at room temperature, both at 0, 2, 4, 8, 12, and 24 h. over the course of one day and within six consecutive months. The R.S.D was also taken as the measure for testing stability and repeatability. A recovery test was conducted to evaluate the accuracy of this method. Each sample was analyzed in triplicate. The total amounts of each compound were calculated from the corresponding calibration curve. The results were given in Table 4.

**Table 4:** Recovery of the three compounds in the *S. miltiorrhiza Bunge* reference extractive.

Analyte	Original mean (mg)	Spiked mean (mg)	Detected mean (mg)	Recovery mean (mg)	RSD (%, *n* = 6)
Cryptotanshinone	1.72	1.70	3.39	98.35	0.67
Tanshinone I	1.05	1.05	2.12	101.57	0.89
Tanshinone IIA	1.95	1.96	3.94	101.59	0.57

All of these findings indicated that the established quantification method was suitable for the quantitative analysis of the three compounds in the reference extractive. The results, listed in Table 5, showed that the total mean content of the three compounds in the reference extractive exceeds 90% and that the RSD was less than 2%.

**Table 5:** Contents of the three compounds contained in the *S. miltiorrhiza Bunge* reference extractive.

Analyte	Content (%, *n* = 3)			Mean Content (%)	RSD (%)
Cryptotanshinone	34.3666	34.5023	34.2292	34.3660	0.40
Tanshinone I	21.0120	21.3019	20.7226	21.0122	1.38
Tanshinone IIA	39.0130	39.3001	38.7278	39.0136	0.73
Total Amount	94.3903	95.1043	93.6796	94.3914	0.75

### 2.7. Determination of the 3 Marker Compounds in Crude Samples Using the Reference Extractive

HPLC was performed using the parameters as mentioned above. The RSD results for the precision, repeatability and stability of the three compounds are listed in Table 6, and the overall RSD was less than 2%. In Table 7, the results of the recovery test indicated that the established method was sufficiently accurate for the determination of the three bioactive constituents in crude drugs.

**Table 6:** Regression, precision, repeatability and stability of the three compounds in *S. miltiorrhiza Bunge.*

Analyte	Regression equation	*r^2^*	Linear range (*μ*g/mL)	Precision (RSD, %, *n* = 6)	Repeatability (RSD, %, *n* = 5)	Stability (RSD, % *n* = 8)
Cryptotanshinone	Y = 30821.89X + 10.63	0.9999	1.75~45.56	0.02	1.51	0.13
Tanshinone I	Y = 15940.54X + 1.02	0.9999	0.99~28.86	0.02	1.46	0.15
Tanshinone IIA	Y = 44870.84X + 0.99	0.9999	1.86~48.18	0.02	1.57	0.11

**Table 7:** Recovery of the three compounds in *S. miltiorrhiza Bunge.*

Analyte	Original mean (mg)	Spiked mean (mg)	Detected mean (mg)	Recovery mean (mg)	RSD (%, *n* = 6)
Cryptotanshinone	227.55	240.57	469.70	100.66	0.84
Tanshinone I	207.74	147.07	355.12	100.21	1.72
Tanshinone IIA	283.35	273.09	557.70	100.46	0.92

### 2.8. Comparison of the Determination of the Three Marker Compounds in the Crude Samples Using Both Reference Extractive and Reference Standards

Based on the above-optimised HPLC conditions, both the reference extractives and standards were simultaneously used in the quantitative determination of seven batches of *S. miltiorrhiza Bunge* samples purchased from representative regions of China. Each sample was analysed in duplicate to obtain the mean content of the three marker compounds. Similar results were obtained by each method. The quantitative results were compared in Table 8, which showed that the reference extractive method was reliable and consistent with that of the reference standards.

In addition, the cost of the preparation of reference extractives, including the cost of crude herbs, reagents and other experimental materials, was also computed and summarised. According to our investigation, about 150 mg reference extractive of *S. miltiorrhiza Bunge* containing 58.53 mg of tanshinone IIA, 31.52 mg of tanshinone I, and 51.56 mg of cryptotanshinone was obtained from 250 g of crude drugs, at a cost of approximately 21 h. of time and RMB 207.58. In comparison, obtaining the three reference standards takes approximately 58 hr. As depicted in Table 9, the cost of the reference extractive was much lower than that of any single reference standard. Furthermore, the method developed by our lab to prepare the *S. miltiorrhiza Bunge* reference extractive can be easily scaled to 100 g.

**Table 8:** Contents of the three compounds in *S. miltiorrhiza Bunge* as determined using reference extractive and standards.

Samplenumber	Batchnumber	Source	Place of origin	Analyte	Mean Content (%, *n* = 3)		RSD (%)
					1 ^a^	2 ^b^	
1	110902002	Beijing Qiancao Zhongyao Yinpian Co., Ltd.	Shandong	Cryptotanshinone	0.1483	0.1480	0.15
				Tanshinone I	0.1384	0.1442	2.90
				Tanshinone IIA	0.1832	0.1802	1.17
2		Beijing Tong Ren Tang Group Co., Ltd.	Shandong	Cryptotanshinone	0.0904	0.0902	0.16
	100255051			Tanshinone I	0.1163	0.1212	2.92
				Tanshinone IIA	0.1745	0.1717	1.14
3		Beijing Tong Ren Tang Group Co., Ltd.	Shandong	Cryptotanshinone	0.0983	0.0981	0.14
	100255050			Tanshinone I	0.1291	0.1345	2.90
				Tanshinone IIA	0.1688	0.1661	1.14
4		Shuang Qiao Yanjing Sliced Medicinal Herbs Factory	Shandong	Cryptotanshinone	0.2125	0.2121	0.13
	1201039			Tanshinone I	0.2196	0.2288	2.90
				Tanshinone IIA	0.2851	0.2805	1.15
5		Beijing Ren Wei Sliced Medicinal Herbs Factory	Anhui	Cryptotanshinone	0.0749	0.0748	0.13
	11072401			Tanshinone I	0.0675	0.0703	2.87
				Tanshinone IIA	0.0879	0.0865	1.14
6		Beijing Fengtai Jinyuan Zhongyao Yinpian Co., Ltd.	Hebei	Cryptotanshinone	0.0719	0.0718	0.14
	11111701			Tanshinone I	0.0697	0.0726	2.89
				Tanshinone IIA	0.0971	0.0955	1.17
7	11100901	Shandong Province, China	Shandong	Cryptotanshinone	0.0446	0.0445	0.22
				Tanshinone I	0.0397	0.0413	2.79
				Tanshinone IIA	0.0627	0.0617	1.14

^a^ Determination with single reference standards; ^b^ Determination with reference extractive of *S. miltiorrhiza Bunge*.

**Table 9:** Preparation costs for the reference extractive and single reference standards.

Specific items		Amount (mg)	Expenses (RMB)	Cost price (RMB/mg)
Single reference standards a	Cryptotanshinone	20	147	7.35
	Tanshinone I	20	179	8.95
	Tanshinone IIA	20	173	8.65
Reference extractive of *S. miltiorrhiza Bunge*		150	207.58	1.38

^a^ The price of the single reference standards is based on the National Institute for Food and Drug Control (NIFDC, Beijing, China) cost.

## 3. Experimental

### 3.1. Instrumentation and Chromatographic Conditions

A Shimadzu LC-20AT HPLC system consisting of a SIL-20A auto-sampler, quaternary pumps and SPD-M20A diode array detector (Shimadzu Corporation, Kyoto, Japan) was used to acquire chromatograms and UV spectra. Chromatographic analysis was conducted on a suitable guard column (Diamons C_18_, 250 mm × 4.6 mm, 5 μm), and HPLC separation was performed using an isocratic elution with aqueous phosphoric acid (0.02%, v/v) and acetonitrile as the mobile phase and a flow rate of 1.0 mL/min. The column temperature was maintained at 25 °C, and the sample injection volume was 10 μL. The DAD detector was set to scan from 200 nm to 800 nm, and 270 nm was selected as detection wavelength for HPLC analysis.

### 3.2. Solvents and Chemicals

HPLC-grade methanol (Merck KGaA, Darmstadt, Germany), acetonitrile (Merck) and deionised water obtained from a Milli-Q water system (Millipore Corp., Bedford, MA, USA) were used to prepare the mobile phase. All solvents were degassed by ultrasonication and an on-line degassing system. Analytical-grade methanol, ethyl acetate, chloroform and acetone (Beijing Chemical Works, Beijing, China) were used for sample preparation.

### 3.3. Reference Standards

Standard samples of tanshinone IIA (Batch number. 110766-200619), cryptotanshinone (Batch number. 110852-200806) and tanshinone I (Batch number. 1428-070322) were purchased from the National Institute for Food and Drug Control (NIFDC, Beijing, China). The purity of each standard was determined to be above 98% by normalization of the peak area detected by HPLC-DAD. The reference extractive, containing 34.37% cryptotanshinone, 21.01% tanshinone IIA and 39.01% tanshinone I, was prepared from the dried roots of *S. miltiorrhiza Bunge* in our laboratory.

### 3.4. Preparation of the Reference Extractive

Medicinal materials (125 g) were subjected to extraction in refluxing acetone (8 times the amount) for 2 h. Crude extract solution (750 mL) was evaporated under reduced pressure to recover the solvent, and then uniformly mixed with 2 g silica gel (160~200 mesh, Qingdao Haiyang Chemical Co., Ltd, Qingdao, China). The mixed powder was evaporated again to dryness, pulverized and chromatographed on a silica gel column (column volume: 50 mL) with petroleum ether- ethyl acetate system as the eluting solvent. Following stepwise elution with petroleum ether-ethyl acetate (25:1, v/v, 4.5 BV) to get rid of the non-target components and chloroform-methanol (10:1, v/v, 3 BV) the total reference extractive solution was obtained. The concentrated solution was further purified by preparative reversed-phase HPLC on a C_18_ column (YMC-Pack-ODS-A, 150 mm × 4.6 mm, 5 µm) with isocratic elution with aqueous methanol (77%, v/v) solution to afford the reference extract with included a mixture of tanshinone IIA, tanshinone I, and cryptotanshinone. The flow rate was 10 mL/min, the injection volume was 1 mL and the detection wavelength was 270 nm.

### 3.5. HPLC-ESI-IT-MS^n^ Analysis of the Reference Extractive

HPLC was performed using an Agilent 1200 system (Agilent Technologies, Santa Clara, CA, USA) equipped with an Diamons C_18_ Column (4.6 mm × 250 mm, 5 μm). The mobile phase consisted of (A) water and (B) ACN and the optimized eluting condition was using an isocratic elution with 42% A. The sample was eluted at 1 mL/min, and the HPLC eluent was split and introduced into the mass spectrometer and DAD detector. The detection wavelength was set in the range 190–400 nm (monitoring wavelength at 270nm).

Mass spectrometry was performed on a 6320 ion trap LC/MS system (Agilent Technologies) equipped with an electrospray ionization source as interface. The MS spectra were acquired over the *m/z* 100–500 range in positive mode. The mass spectrometer condition were as follows: drying gas temperature, 320 °C; drying gas (N_2_) flow rate, 10 L/min; nebulizer, 42 psi; capillary exit voltage, 101.5 V; skimmer voltage, 40 V; Oct 1 DC, 12 V; Oct 2 DC, 1.7 V. All the mass data was processed with the accurate Data Analysis for 6300 series ion Trap LC/MS Version 4.0 Chemstation (Agilent Technologies).

### 3.6. Samples Collection and Preparation

*S. miltiorrhiza Bunge* was collected from different commercial herb suppliers located in China. All of the samples were authenticated by Professor Cheng Ming of the Institute of Chinese Materia Medica, Chinese Academy of Chinese Medical Sciences, based on the description in the *Chinese Pharmacopoeia* (2010 edition). The sources and origin of the samples, including batch numbers, are listed in Table 8. 

Regarding the preparation of the test solutions of the crude drugs, comparative studies on ultrasonic and reflux extraction were implemented using the contents of the three marker compounds as the main indicators. The ultrasonic treatment procedure was found to be the best extraction method, and the preparation of the test solution was carried out as described above. *S. miltiorrhiza Bunge* powder (0.30 g) was extracted with methanol for 30, 45, 60 and 90 min using ultrasonication and it was suggested that all three compounds were almost completely extracted within 45 min. The reference standards were prepared by accurately weighing tanshinone IIA (11.64 mg), tanshinone I (10.00 mg) and cryptotanshinone (9.80 mg) into 100 mL, 50 mL and 50 mL volumetric flasks, respectively, and adding methanol to volume. The resulting concentrations of tanshinone IIA, cryptotanshinone and tanshinone I were 116.40, 200.00 and 196.00 μg mL^−1^, respectively, and the solution was filtered through a 0.45 mm PTFE filter (Iwaki Glass, Tokyo, Japan) as the reference solution. Meanwhile, approximately 10.25 mg of the reference extractive was dissolved with methanol in a 50 mL volumetric flask to volume and mixed well. The mixture was also filtered through a 0.45 mm PTFE filter (Iwaki Glass) to obtain the test solution.

## 4. Conclusions

Due to the lack of adequate reference standards in the study of the quality control of TCMs, the conception of the reference extractive was put forward in the *Chinese Pharmacopoeia* (2005 edition) for the first time. In this paper, a rapid and efficient quality assessment method was first established to meet the complex requirements of the quantitative determination of TCMs and then it was successfully applied to the quality control of various *S. miltiorrhiza Bunge* samples. Our results confirmed that using reference extractive to determine drug contents was both feasible and reliable. 

Based on above comparison and analysis, the preparation of the reference extractive was generally easier than that of the single reference standard, which helped to reduce substantially the analysis cost. Moreover, in comparison to the single reference standard, the reference extractive used for quantitative analysis had advantages of controlling multi-index constituents and improving the specificity of identification. Although some problems, such as the source of herbs used for preparation of the reference extractive, selection of compositions contained in the reference extractive, standardization of the reference extractive preparation technology, *etc.* should be taken into account for future research in order to enhance its quality, the significance of our results lies in the demonstration that it is feasible and reliable to apply reference extractives for quantitative analysis. Such a method would not only exhibit great superiority in terms of simplicity, time efficiency and economy but also provide a powerful and rational method for the quality evaluation of TCMs. We hope that this study will contribute to future studies improving the quality standard of TCMs.
